# Considerations on the Wear Behavior of Vacuum-Remelted ZrO_2_-Reinforced Self-Fluxing Ni-Based Thermally Sprayed Alloys

**DOI:** 10.3390/ma16145183

**Published:** 2023-07-24

**Authors:** Norbert Kazamer, Roxana Muntean, Ion-Dragoș Uțu, Gabriela Mărginean

**Affiliations:** 1 Westphalian Energy Institute, Westphalian University of Applied Sciences, Neidenburgerstr. 43, 45897 Gelsenkirchen, Germany; norbert.kazamer@w-hs.de; 2 Materials and Manufacturing Engineering Department, Faculty of Mechanical Engineering, Politehnica University of Timișoara, Bvd. Mihai Viteazu nr. 1, 300222 Timișoara, Romania; dragos.utu@upt.ro; 3 Institute of Mechanical Engineering, Westphalian University of Applied Sciences, Neidenburgerstr. 43, 45897 Gelsenkirchen, Germany; gabriela.marginean@w-hs.de

**Keywords:** self-fluxing, ZrO_2_, NiCrBSi, vacuum post-treatment, thermal spraying

## Abstract

Without proper post-processing (often using flame, furnace, laser remelting, and induction) or reinforcements’ addition, Ni-based flame-sprayed coatings generally manifest moderate adhesion to the substrate, high porosity, unmelted particles, undesirable oxides, or weak wear resistance and mechanical properties. The current research aimed to investigate the addition of ZrO_2_ as reinforcement to the self-fluxing alloy coatings. Mechanically mixed NiCrBSi-ZrO_2_ powders were thermally sprayed onto an industrially relevant high-grade steel. After thermal spraying, the samples were differently post-processed with a flame gun and with a vacuum furnace, respectively. Scanning electron microscopy showed a porosity reduction for the vacuum-heat-treated samples compared to that of the flame-post-processed ones. X-ray diffraction measurements showed differences in the main peaks of the patterns for the thermal processed samples compared to the as-sprayed ones, these having a direct influence on the mechanical behavior of the coatings. Although a slight microhardness decrease was observed in the case of vacuum-remelted samples, the overall low porosity and the phase differences helped the coating to perform better during wear-resistance testing, realized using a ball-on-disk arrangement, compared to the as-sprayed reference samples.

## 1. Introduction

The thermal-spraying technology is a convenient method to process powder metallic materials to a coating, as these are deposited at elevated temperatures in a partially or totally molted state by overlapping material droplets impacted onto a substrate surface [1]. The flattening of the droplets, the rapid cooling, and their solidification transform the melted powder into a performant coating. Thermally sprayed Ni-based powders are well-known materials for applications that require wear and corrosion resistance, such as turbine blades, rotary tillers, boiler tube steel components [2], or cutting blades [3]. Even if the characteristics of the coatings are unique, the Ni-based system requires permanent improvements to keep up with industrial development. Since the content of the chemical elements cannot be varied indefinitely to improve the alloy or the resulting coatings, other developments in terms of novel deposition techniques, post-processing, or the introduction of reinforcing particles have been proposed. Although new methods, such as one-step atmospheric plasma spraying deposition [4] or powder laser cladding with no further post-processing, were recently reported [5], most of the state-of-the-art Ni-based self-fluxing alloy coatings need further remelting [6,7,8,9]. Post-processed coatings show improvement in fracture toughness and overall improved mechanical performance [10], as well as an increase in wear and corrosion resistance [11]. Modifications of coating density, improvement of the inter-splat bonding, and adhesion strength to the substrate are aspects also reported in the literature [12]. Various remelting technologies have been recently investigated, such as flame [13,14], laser [15,16,17], plasma [18,19], or furnace treatment [6,15,20].

Although Ni- and Co-based self-fluxing alloy coatings already constitute adequate alternatives to, e.g., chromium and cobalt-based cermets, which are known for their harmful effect on the environment [21], the material development is constantly pushed through different added reinforcements. WC or WC-Co are classic materials that are frequently added as a reinforcement, helping the cavitation resistance [22] or increasing the wear behavior, as they may change the wear mechanism from adhesion to abrasion and fatigue wear [5], respectively. Dey et al. [23] recently reported the addition of Ag and WS_2_ as solid lubricants to the matrix, achieving a coefficient of friction as low as 0.25 and very low wear rates, the findings being of high industrial relevance. Molybdenum has been recently employed as a reinforcing material for plasma-sprayed NiCrBSi for brake disc coatings [19]. Kılıç et al. reported that the addition of ductile phases to the brake disc coatings was found to be beneficial for the coefficient of friction reduction and for an overall wear resistance improvement [19]. SiO_2_ tribo-films formed on SiC-reinforced Ni-based coatings have also been described to greatly improve the boundary lubrication effect [24,25]. Wang et al. recently reported a comprehensive study regarding the effect of heat treatment at different temperatures on the microstructure of NiCrBSi-TiN composite coatings [6], while Huang et al. successfully used TiC as strengthening particles [26]. Recently, our research group reported on titanium-reinforced coatings and observed that the TiB_2_ particles acted as physical barrier against wear and corrosion phenomena [27,28].

ZrO_2_ is a ceramic compound with some exceptional properties. It exhibits three temperature-dependent polymorphs: monoclinic, tetragonal, and cubic. Each of them has different advantages and disadvantages, but the most valuable are the high-temperature phases, tetragonal and cubic. In order to stabilize these two specific phases at room temperature, ZrO_2_ can be doped during synthesis with lower-valence oxides such as MgO, Y_2_O_3_, or CaO [29,30]. By far, the most used combination is the one with yttrium oxide [31,32,33,34,35], showing industrial employment in high-temperature aerospace gas turbine engines [36,37]. Studies regarding the effect of nanostructured zirconia additions on the microstructure, micro-hardness, and wear performance of nickel-based hard-facing alloys deposited onto low-carbon steel via atmospheric plasma spraying were also reported. It was concluded that the hardness and wear behavior of coatings containing ZrO_2_ were improved, while the friction coefficient decreased [38]. More recently, a solution of incorporating La oxides in different stoichiometry was found to be beneficial for thermal barrier applications [39,40,41]. For biomedical purposes, a Co-Cr alloy material was coated with different contents of HA-ZrO_2_ mixtures, using the flame spraying method. The effect of HA/ZrO_2_ particles on the corrosion resistance of the resulting coatings was evaluated, and the reported results highlighted a significant improvement in the corrosion behavior of the bio-ceramic-reinforced coatings [42]. Moreover, ZrO_2_ shows impressive toughness, strength, and fracture strength; low elasticity module and fatigue; and wear resistance [30,43].

Due to the previously mentioned very attractive properties, the present study focused on the development of NiCrBSi-ZrO_2_ thermally sprayed and flame/vacuum-remelted coatings. The suggested approach should provide improved results in terms of wear resistance, enhanced by the presence of ceramic-reinforcement particles correlated with the effect of the thermal post-treatment. After mechanically mixing the powder, the coatings were deposited by flame spraying and further post-processed either with a flame torch or in a vacuum furnace. The effect of the ZrO_2_ addition on the microstructure, phase composition, microhardness, and wear behavior of the resulting composite coatings was investigated, and the main findings are reported in correlation with the microstructural evolution.

## 2. Materials and Methods

### 2.1. Materials and Coating Deposition

**Figure 1:**
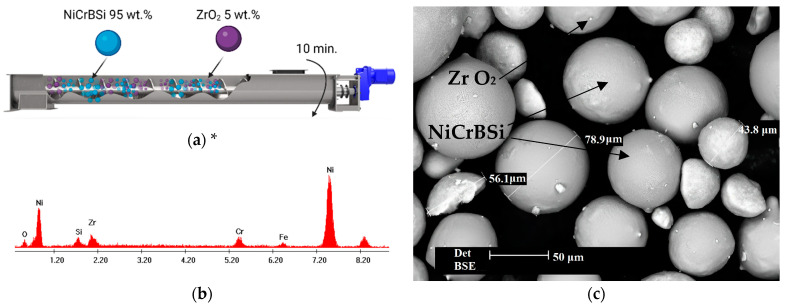
(**a**) The schematics of the powder mixing process in the ribbon powder mixer, (**b**) the chemical composition, and (**c**) the morphology of the mixed powders (* created with BioRender.com).

The materials involved in the current study are a self-fluxing atomized powder with the chemical composition Ni-6Cr-1B-4Si-1.5Fe-0.3C and grain size of −60 + 100 μm, available from LSN Engineering GmbH, Herzogenrath, Germany; and yttrium oxide partially stabilized zirconia (PSZ) powder in tetragonal predominant structure, without inner porosity and a finer particle size distribution of around −5 + 25 μm, which is a commercial product from the Kläger Spritzguss GmbH & Co. KG, Dornstetten, Germany. Since the monoclinic ZrO_2_ phase presents poor mechanical properties associated with a volume expansion during the cooling process (due to the tetragonal-monoclinic transition), it is not preferred in applications that require high mechanical strength. Using Y_2_O_3_ as a stabilizing agent enables the production of ceramic ZrO_2_ metastable tetragonal grains. The feedstock powder is, therefore, a blend between the matrix NiCrBSi and the reinforcement powder, ZrO_2_, with a weight ratio of 95:5, mixed with the aid of a handheld ribbon powder mixer. The mixing process, which is schematized in Figure 1a, relies on the principle of manually rotating the apparatus in a controlled manner for 10 min while having the powders placed inside the assembly. The ribbon powder mixer inherently ensures that the two materials are uniformly combined, while the blending of the powder through the rotating spiral eliminates the risk of percolation (segregation) of the smaller ZrO_2_-reinforcing particles. Although it can be considered that the mixing process does not affect the chemical composition of the material, a small change regarding this aspect can be observed. The energy-dispersive X-ray spectrum (EDX) of the mixed powder (Figure 1b) reveals the chemical composition of the matrix, as well as the elements Zr and O from the reinforcement powder, this being a consequence of the material friction during the mechanical mixing, due to a minor detachment of the microparticles from each powder. The scanning electron micrograph (SEM) presented in Figure 1c clearly shows the spherical shape of the NiCrBSi powder and confirms the size distribution provided by the producer.

The powder deposition was performed on an S355JR structural steel substrate with the aid of a Methaterm MPP-85 (5PII Type) oxyacetylene gun, at the partner company Karl Schumacher GmbH, Bochum, Germany. Before deposition, the substrate material, which was cut into 200 mm × 100 mm × 10 mm coupons, was grit-blasted and heated up to 105 °C to assure the proper mechanical interlocking of the subsequently deposited coating. The applied deposition parameters and methodology were optimized and described in a previous study on similar NiCrBSi feedstock powders but with different reinforcing materials [27]. The feed rate was 2.5 kg h^−1^; the stoichiometry of the gases was 1:2 for C_2_H_2_:O_2_, generating an oxidizing flame, and the stand-off distance was 120 mm. The temperature of the flame was measured at 80 mm from the nozzle and was kept at ≈2850 °C by maintaining a constant gas flow rate.

### 2.2. Post-Processing

**Figure 2:**
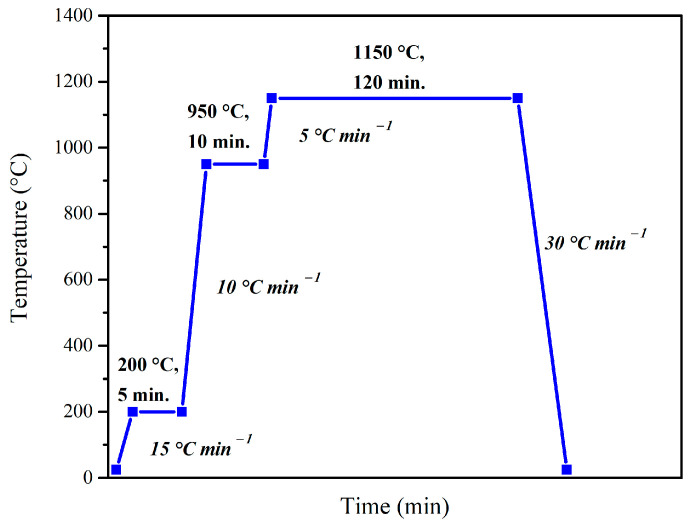
The vacuum-heat-treatment process program applied for the NiCrBSi-ZrO_2_ flame-sprayed coatings.

After the thermal spraying process, two different post-treatments were considered. The vacuum treatment was performed at 10^−2^ Pa in a HITERM 80-200 cold-wall (water-cooled) vertical vacuum furnace (Hitec Material Group, Shandong, China) equipped with a turbomolecular and an oil-sealed pump and controlled with the iTools Engineering software (v9.73). The post-treatment program schematized in Figure 2 shows the three-interval heating stages applied during the vacuum treatment. The first one, at 200 °C, is extremely beneath the melting range of the powder but high enough for material soaking. Slow heating was performed afterward with 5 °C min^−1^ to prevent internal stress occurrence. The first holding at 950 °C for 10 min offered enough time for pressure-level stabilization inside the furnace. This temperature is under the melting range of the NiCrBSi matrix but is high enough for an additional soaking of material. The final holding temperature at 1150 °C was chosen after taking into account the melting range of the feedstock powder that was previously determined for this chemical composition by using differential scanning calorimetry [28]. The holding time of 120 min at 1150 °C generates an equalization of the temperature throughout the furnace chamber, enables a complete remelting of the self-fluxing alloy, and promotes the mandatory fluidity required for the densification of the coatings. Moreover, during the heat treatment in the vacuum furnace, phenomena such as degassing or decomposition of previously formed oxides may occur. The cooling ramp was performed with a high enough rate of 30 °C min^−1^ to prevent unnecessary grain growth in the coating but slow enough to avoid internal stresses.

The flame remelting was performed with the same deposition gun, at 1150 °C, for 120 min, at the identical working parameters reported for the deposition itself.

### 2.3. Characterization Methods

The microstructural investigation, combined with the elemental analysis performed on the feedstock powder and on the thermally sprayed coatings, was carried out with a scanning electron microscope (SEM), Philips XL30 ESEM, Eindhoven, The Netherlands, equipped with an energy-dispersive X-ray spectrometer (EDX), Ametek, Berwyn, PA, USA, at a 10 mm working distance and a 25 kV cathode voltage. The phase composition was determined by corroborating the information from the EDX analysis with the data obtained from the X-ray diffraction (XRD), Philips X’Pert MDP X-ray diffractometer, Malvern PANalytical, Malvern, UK, acquired at 40 kV and 40 mA, at a scan rate of 0.01° 2θ min^−1^, at an angular resolution of 5 × 10^−3^ 2θ, using Cu-Kα radiation. The porosity was calculated in the ImageJ software (a free open-source Java-based program, v.1.54d) by processing the SEM micrographs, according to ASTM E2109-01 [44] standard. The cross-section of the thermally sprayed coatings was obtained by conventional metallographic preparation, according to the ASTM E1920-03 [45], consisting of grinding with 180, 320, and 800 grit SiC abrasive paper, followed by rough polishing with diamond suspension (3 and 6 μm).

The coefficient of friction (COF) was measured using a ball-on-disc arrangement on a tribometer, TRB, CSM Instruments, Switzerland, at a linear speed of 15 cm s^−1^, an acquisition rate of 10 Hz, and a total load of 10 N, in normal atmosphere, at room temperature, without lubrication, according to ASTM G99-17 [46]. The stop condition was set to 10^3^ m of total worn distance, with the requirement that the material should reach a steady-state condition. WC-Co-6 mm diameter balls were used as a counter body. The generated wear depth of the interacting sample surface and the ball was measured with a confocal VK-X260K laser scanning and a digital VHX-600 microscope, both from Keyence International, Belgium. The HV0.3 microhardness indentations were performed in cross-section for at least 5 points, with a microhardness tester, ZHVµ-M, ZwickRoell AG, Ulm, Germany, and the average values were considered. To assure the reproducibility of the results, three measurement repetitions were performed on each specimen.

## 3. Results and Discussion

**Figure 3:**
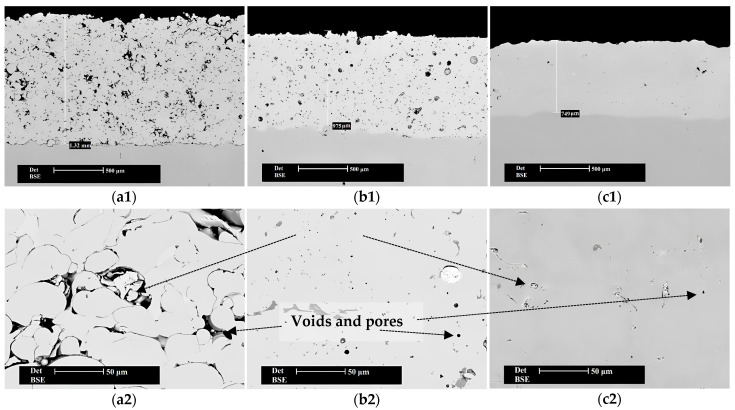
Cross-section micrographs of the (**a1**,**a2**) as-sprayed, (**b1**,**b2**) flame, and (**c1**,**c2**), vacuum-remelted coatings.

The cross-section micrographs from Figure 3 present the as-sprayed and the remelted specimens, displaying the whole thickness of the coating, as well as the detailed microstructure, where regions of interest were further magnified. The as-sprayed sample presents a typical morphology of a thermal sprayed coating, with a strong inhomogeneous structure, having partially melted and unmelted particles, microcracks, high surface roughness, and an estimated porosity of over 10%, attributed to the layer-by-layer coating formation during the deposition process (interlamellar porosity). The homogeneity and compactness of the coating are improved, while the porosity decreases when the flame and the vacuum remelting treatment are applied. A smaller number of defects and voids are found for the vacuum-remelted sample, where a porosity of around 2% is reached, compared to the 4% for the flame-remelted one. The lower porosity value obtained for the vacuum-remelted sample is justified by the self-fluxing character of the NiCrBSi alloy that favors the densification, correlated with the high-vacuum atmosphere, which promotes a complete degassing process. Moreover, once the remelting is applied, a decrease in the coating thickness can also be observed. During the thermal post-processing, the trapped gases inside the coating are released, tightening the coating and, consequently, decreasing its thickness. From the initial value of 1300 μm obtained for the as-sprayed coatings, the densification that occurred during the remelting process led to a final thickness of around 975 μm for the flame-remelted coating and 750 μm in the case of the vacuum-remelted sample. The latter one has a reduced thickness compared to that of the flame post-processed specimen since the exposure time at a higher temperature was longer, the controlled cooling in the furnace clearly took an extended time, and the vacuum played a significant role in the degassing step. While the coating–substrate interface presents minor cracks and poor adhesion in the case of as-sprayed coatings, once the post-treatment is applied, this aspect is significantly improved. The SEM micrographs from Figure 3 acquired at higher magnifications reveal the ZrO_2_ particles placed in the voids left in the matrix during the deposition step. Some regions of reinforcing ZrO_2_ agglomerates were also found in the vacuum-remelted samples due to the insufficient mixing of the powders. The post-treatment, in both cases, eliminated the splat boundaries observed in the case of as-sprayed coatings, as well as the majority of defects that occurred during the deposition process. 

**Figure 4:**
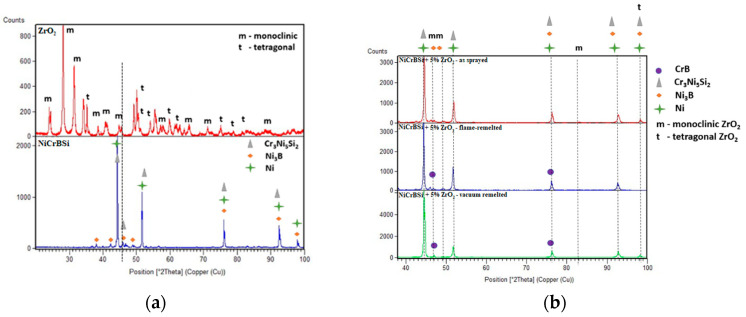
XRD patterns of the (**a**) powders and (**b**) coatings in different states.

The XRD analysis, which is displayed in Figure 4, indicates, in the case of the ZrO_2_ reinforcement material, the presence of mixed monoclinic (JCPDS no. 81-1314), and tetragonal (JCPDS no. 81-1544) phases, as also reported in other studies [47]. When considering the full-width-at-half-maximum intensity for the most intense three peaks of the ZrO_2_ powder (FWHM), according to the Scherrer equation, it can be concluded that the tetragonal phase presents higher crystallite sizes compared to the monoclinic phase. From the reflections at 50.2, the average tetragonal ZrO_2_ crystallites were estimated to be about 67 nm, while the crystallites corresponding to the monoclinic phase, at 28.13 and 31.42 reflections, did not exceed 50 nm. The matrix aligns well with the classical phase interpretation for the Ni-based self-fluxing coatings [9,28,48,49] with a Ni-predominant phase and a σ Cr_3_Ni_5_Si_2_ intermetallic compound. The coating’s post-processing led to the formation of hard CrB or Ni_3_B phases, whose presence is beneficial for the wear resistance of the coatings [11,22,28,50].

The presence of ZrO_2_ phases in monoclinic and tetragonal states in the XRD patterns of the coatings suggests that the powders were stable during the thermal processing since the typical temperature for monoclinic to tetragonal transformation (1170 °C) was avoided, especially during the remelting process.

**Figure 5:**
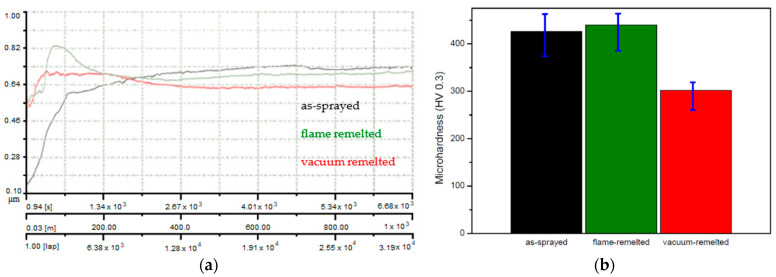
(**a**) Coefficient of friction and (**b**) HV0.3 microhardness of the (black) as-sprayed, (green) flame, and (red) vacuum post-processed samples.

Generally, the coefficient of friction is not necessarily in direct correlation with the wear rate of a part, but for specific industries, e.g., brake discs [19], the COF is the basic phenomenon that needs to be analyzed first. The variation of the COF commonly depends on several parameters of the analyzed system, as well as on the interfacial conditions between friction partners, such as load, geometry, sliding velocity, surface roughness, or lubrication. Analyzing Figure 5a, it can be observed that the rapid initial increase of the COF values may be attributed to the micro-convex shape of the counter bodies in contact with the flat surface of the samples. Once the contact becomes smooth, the overall friction starts to become balanced, and the sample starts to move toward a steady state. Although the coatings behave similarly, the vacuum post-processed sample reached, nevertheless, the smallest average COF value, stabilizing at 0.63, while the as-sprayed and the flame-remelted samples remained close to each other at 0.7 and 0.68, respectively. In this situation, the COF appears to be independent of the applied remelting technology. The present values of the COF are in good agreement with the ones of Weicheng et al. [51] and Gahr et al. [52], both studies testing sliding wear in unlubricated conditions for ZrO_2_-reinforced materials.

The microhardness of the coatings graphically displayed in the bar chart from Figure 5b shows that the highest values were obtained for the flame-remelted and the as-sprayed coatings, followed by the vacuum-remelted one. The microhardness slight increase in the case of the flame-remelted coatings compared to the as-sprayed samples may be attributed to the reduction of porosity and the enhancement of intersplat cohesion. It can be assumed that, in the case of samples treated in the vacuum furnace, the grains did not develop improved hardness due to the lower cooling rate, compared to the flame-remelted coatings, which were spontaneously cooled (at a higher cooling rate). The decrease in hardness is typical for the vacuum and furnace remelting process, and similar behavior has been also reported [11]. Nevertheless, compared to the other investigated coatings, the latter one presents the lowest number of defects, being the least vulnerable to phenomena such as corrosion or wear. Although lower, the microhardness of the vacuum-processed coating has a minor standard deviation between the measurements and, hence, a more homogeneous phase distribution and presumably lower values of internal stresses.

**Figure 6:**
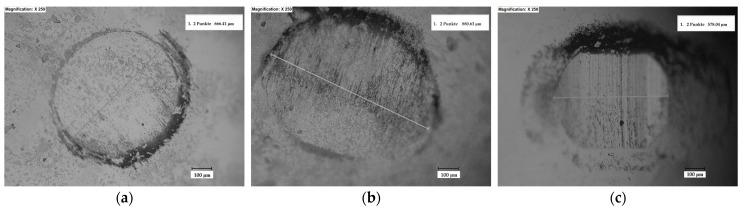
Micrographs of the worn region of the WC-Co static counter body tested against the (**a**) as-sprayed, (**b**) flame, and (**c**) vacuum-furnace post-processed samples after pin-on-disc test.

**Figure 7:**
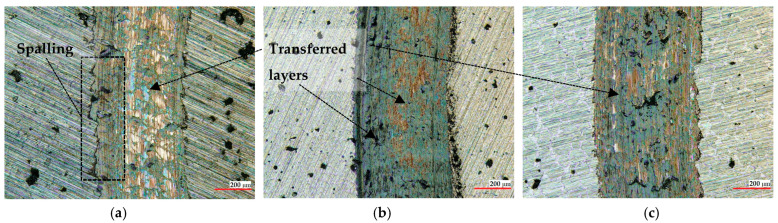
Micrographs of the worn regions of the (**a**) as-sprayed, (**b**) flame, and (**c**) vacuum furnace post-processed samples tested against the WC-Co counter body after the ball-on-disc tests.

**Figure 8:**
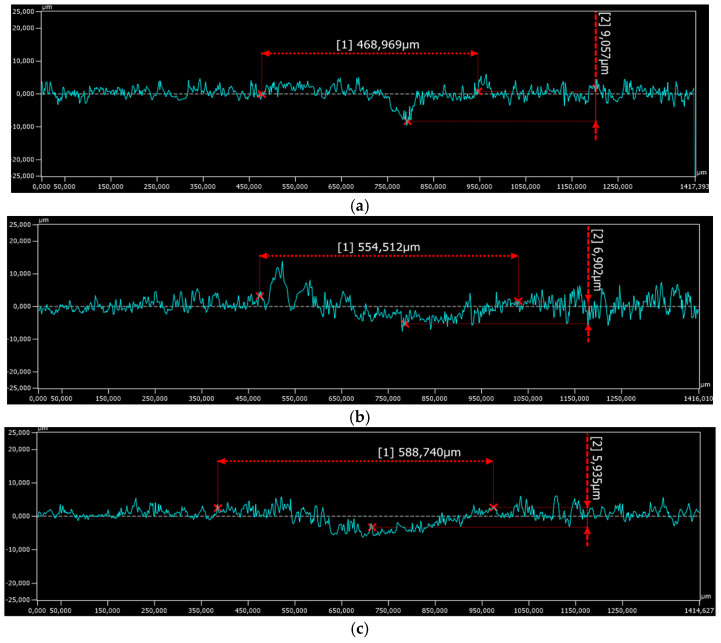
Wear track depth on the (**a**) as-sprayed, (**b**) flame, and (**c**) vacuum-remelted coatings.

The wear tracks of the coatings and of the reciprocal counter bodies after the ball-on-disc measurements are displayed in Figure 6, Figure 7 and Figure 8, and they show similar widths and depths and, hence, comparable wear rates and the same wear mechanisms for all the coatings. As a general remark, no delamination or brittle fracture, commonly produced in thermal sprayed coatings, can be observed on the worn surfaces. Debris with a thickness of a few micrometers transferred to the surface of the coatings can be clearly distinguished in Figure 7. The phenomenon of transferred layers can be noticed for all three samples, with the smallest amount being present on the coating post-processed in the vacuum furnace. This might be attributed to the strong densification during the controlled atmosphere post-treatment, where trapped gases inside the coating were heavily eliminated, and moreover, due to the presence of ZrO_2_, which might generate an auto-lubricating film, as already observed by other researchers [53,54]. The presence of transferred layers, as reported in previous studies, favored the achievement of a steady state on the COF evolution, and a similar trend is observed here as well [51,55]. Furthermore, the as-sprayed sample showed large regions of spalling where supposedly even the matrix was removed, the occurrence of such phenomena emphasizing the importance of the post-treatment. The spalling phenomenon is frequently attributed to the presence of defects such as porosity or non-uniform phase distribution [56]. A large surface of the worn track, in the case of the as-sprayed coatings, presents regions with spalling, and this has a negative influence on the wear behavior. The friction process generated a significant amount of abrasive debris that was removed, causing furrows, indicating a typical kind of abrasive wear.

**Figure 9:**
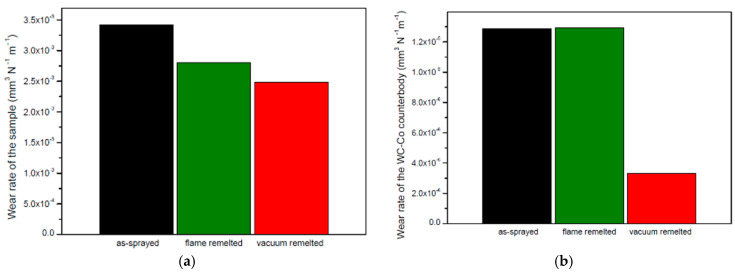
Wear rates of the tested (**a**) samples and (**b**) WC-Co counter bodies.

The specific wear rates of the samples and of the counter bodies were calculated according to the Archard Equation [57]. As presented in Figure 9, the wear rates of the coatings gradually decreased when a thermal post-treatment was applied. In the case of flame-remelted samples, the wear rate was reduced by almost 25%, compared to the as-sprayed coatings, while the vacuum treatment had a more intense effect on the wear-rate reduction. Analyzing the counter bodies, it can be remarked that the one used against the vacuum-treated sample presented the lowest wear rate, while for the as-sprayed and the flame-remelted counter bodies, similar values were observed. 

## 4. Conclusions

Successful NiCrBSi-ZrO_2_ samples with a ratio of 95:5 wt.% were prepared by oxyacetylene flame spraying and post-processed by a flame torch and a vacuum furnace treatment. The microstructural investigations showed a roughness decrease and a homogeneity increase for the flame and vacuum-remelted coatings, compared to the as-sprayed samples. This aspect is owed to the densification achieved during the thermal post-treatment when trapped gases were gradually released from the coatings. Some regions of ZrO_2_ agglomerates were observed in the coatings, but their number was lower in the case of the vacuum-remelted coating, where a longer exposure time at high temperatures was achieved. No phase transformation of the ZrO_2_ during the high-temperature treatment was observed in the XRD analysis. Furthermore, σ-Cr_3_Ni_5_Si_2_ intermetallic and CrB or Ni_3_B phases were also identified as the main phases for all the investigated coatings.

Ball-on-disc measurements showed a comparable coefficient of friction for all three types of coatings, and the steady-state regime was achieved at similar values. Large regions of spalling on the as-sprayed sample were observed, while on the other two treated samples, transferred layers from the WC-Co counter body were identified. The microhardness of the as-sprayed and flame-remelted coatings was similarly high, while the vacuum-furnace-treated one achieved a smaller value. Nevertheless, the latter one showed a smaller standard deviation, indicating a more homogeneous phase distribution and presumably lower internal stresses, thus making it less vulnerable to corrosion phenomena.

## Data Availability

Not applicable.
